# Non-contiguous finished genome sequence and description of *Paucisalibacillus algeriensis* sp. nov.

**DOI:** 10.4056/sigs.5611012

**Published:** 2014-03-18

**Authors:** Esma Bendjama, Lotfi Loucif, Seydina M. Diene, Caroline Michelle, Djamila Gacemi-Kirane, Jean-Marc Rolain

**Affiliations:** 1 Unité de recherche sur les maladies infectieuses et tropicales émergentes (URMITE), UMR CNRS, IHU Méditerranée Infection, Faculté de Médecine et de Pharmacie, Aix-Marseille-Université, Marseille, France.; 2 Département de Biochimie, Faculté des Sciences, Université Badji Mokhtar, Annaba, Algérie.; 3 Département des Sciences Biologiques, Faculté des Sciences, Université El Hadj Lakhdar, Batna, Algérie.

**Keywords:** *Paucisalibacillus algeriensis*, taxono-genomics, soil, hypersaline environments, flagella

## Abstract

*Paucisalibacillus algeriensis* strain EB02^T^ is the type strain of *Paucisalibacillus algeriensis* sp. nov., a new species within the genus *Paucisalibacillus*. This strain, whose genome is described here, was isolated from soil sample from the hypersaline lake Ezzemoul Sabkha in northeastern Algeria. *Paucisalibacillus algeriensis* is a Gram-positive and strictly aerobic bacterium. Here we describe the features of this organism, together with the complete genome sequence and annotation. The 4,006,766 bp long genome (1 chromosome but no plasmid) exhibits a low G+C content of 36% and contains 3,956 protein-coding and 82 RNA genes, including 9 rRNA genes.

## Introduction

Strain EB02^T^ (= CSUR P858 = DSM 27335) is the type strain of *Paucisalibacillus algeriensis* sp. nov. It is a strictly aerobic Gram-positive rod, motile by means of peritrichous flagella, and spore-forming bacteria. It was isolated from a soil sample from the hypersaline lake Ezzemoul Sabkha of Oum-El-Bouaghi region in northeastern Algeria, which is the largest nesting area of Mediterranean flamingos. This lake is a Ramsar site (http://www.ramsar.org).

The genus *Paucisalibacillus*belongs in the *Bacillaceae*family, and was created by Nunes in 2006 [1]. To date, the genus contains only one validly published species, *Paucisalibacillus globulus*strain B22^T^ which was isolated from potting soil in Portugal [1]. It has been described as a Gram positive rod-shaped bacterium, strictly aerobic, spore-forming and motile by means of two polar flagella at one end. It grows in the absence of NaCl, but a low NaCl concentration (1% w/v) improves growth [1].

The current bacterial taxonomy relies on a combination of various phenotypic, chemotaxonomic and genetic criteria [2-4]. The essential genetic criteria used are DNA-DNA hybridization, which is the ‘gold standard’ criterion to define bacterial species [3,5,6], G+C content and 16S rRNA gene sequence based phylogeny [7]. However, these criteria have several drawbacks and their cutoffs can not be used for all bacterial genera [8]. Presently, as the number of available bacterial genomes is increasing, while costs of whole genome sequencing are decreasing, it has been proposed that genomic information and MALDI-TOF spectra [9] be included with the main phenotypic characteristics of a strain, in a polyphasic approach (taxono-genomics) to the description of new bacterial taxa [8,10-23].

Here we present a summary classification and a set of features for *Paucisalibacillus algeriensis* sp. nov. strain EB02^T^ together with the description of the complete genome sequence and annotation. These characteristics support the circumscription of the species *Paucisalibacillus algeriensis*.

## Classification and features

**Figure 1:**
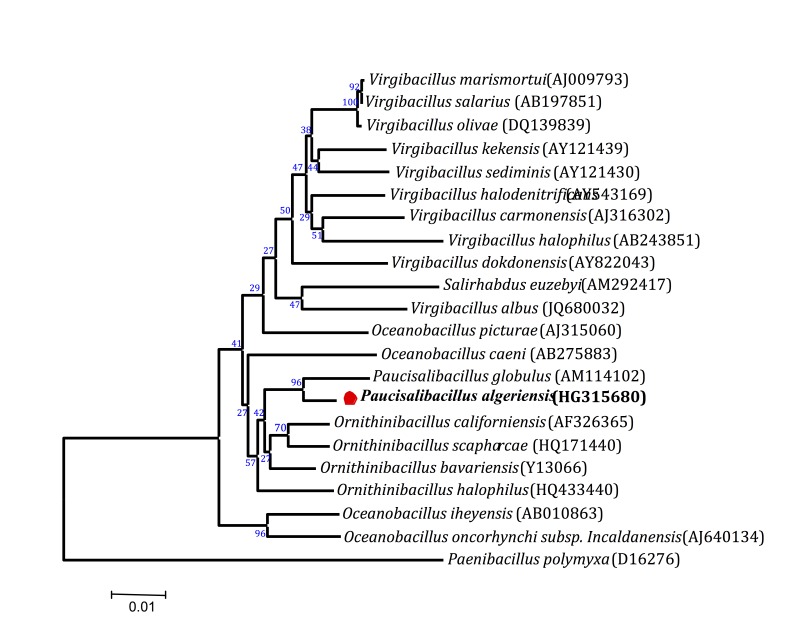
A consensus phylogenetic tree based on 16S rRNA gene sequence comparisons, showing the relationships between strain EB02^T^ *Paucisalibacillus algeriensis* and other type strains from the genera *Paucisalibacillus**,* *Ornithinibacillus**,* *Oceanobacillus**,* *Virgibacillus* and *Salirhabdus**.* GenBank accession numbers are displayed in parentheses. Sequences were aligned using CLUSTALW, and phylogenetic inferences obtained using the neighbor-joining method [35] in the MEGA 5 software package [36]. Numbers above the nodes are percentages of bootstrap values obtained from 1,000 replicates that support the node. *Paenibacillus polymyxa* was used as the outgroup. The scale bar represents 0.01 substitutions per nucleotide position.

*Paucisalibacillus algeriensis* strain EB02^T^ was isolated accidentally in July 2012 during research work for the isolation of halophilic actinomycetes, and further characterized. The source of the isolate was a hypersaline soil sample from the Northwestern periphery of the hypersaline lake Ezzemoul Sabkha in the Oum-El-Bouaghi region of northeastern Algeria. This part of the lake is bounded by halophilic vegetation. Samples were taken aseptically at a depth of 10 cm and transferred to sterile containers, then transported in a cooler (4°C) to our lab in Algeria. 10 g of hyprersaline soil were suspended in 90 ml of sterile saline water (0.9% NaCl) and vigorously vortexed. Tenfold serial dilutions up to10^-5^ of the soil suspension were plated in ISP (International *Streptomyces* Project) medium 2 (dextrose 4 g/l, malt extract 10 g/l, yeast extract 4 g/l, agar 20 g/l) [24] and the plates were incubated at 30°C for 21 days. Strain EB02^T^ was obtained after 24 h of incubation. In order to obtain a pure culture, colonies were transferred after microscopic examination to Nutrient Agar (NA) medium (meat extract 1 g/l, peptone 5 g/l, yeast extract 2 g/l, sodium chloride 5 g/l, agar 15 g/l). *Paucisalibacillus algeriensis* sp. nov. strain EB02^T^ (Table 1) was isolated by cultivation under aerobic conditions at 30°C. When compared to sequences available in GenBank database using the BLAST program through the National Center for Biotechnology Information (NCBI) server, the 16S rRNA gene sequence of *Paucisalibacillus algeriensis* strain EB02^T^ (GenBank accession number HG315680) exhibited the highest identity (98.2%) with *Paucisalibacillus globulus*type strain DSM18846^T^ (Figure 1), the phylogenetically closest validly published *Paucisalibacillus*species. This value was lower than the 98.7% 16S rRNA gene sequence threshold recommended by Stackebrandt and Ebers to delineate a new species without carrying out DNA-DNA hybridizidation [7].

**Table 1:** Classification and general features of *Paucisalibacillus algeriensis* strain EB02^T^

**MIGS ID**	**Property**	**Term**	**Evidence code^a^**
		Domain *Bacteria*	TAS [25]
		Phylum *Firmicutes*	TAS [26-28]
		Class *Bacilli*	TAS [29,30]
	Current classification	Order *Bacillales*	TAS [31,32]
		Family *Bacillaceae*	TAS [31,33]
		Genus *Paucisalibacillus*	TAS [1]
		Species *Paucisalibacillus algeriensis*	IDA
		Type strain: EB02^T^	IDA
	Gram stain	Positive	IDA
	Cell shape	Rod-shaped	IDA
	Motility	Motile	IDA
	Sporulation	Sporulating	IDA
	Temperature range	Between 25°C and 50°C	IDA
	Optimum temperature	30°C-37°C	IDA
MIGS-6.3	Salinity	Growth in LB medium + 0-5% NaCl	IDA
MIGS-22	Oxygen requirement	Aerobic	IDA
	Carbon source	Unknown	NAS
	Energy source	Unknown	NAS
MIGS-6	Habitat	Hypersaline soil sample	IDA
MIGS-15	Biotic relationship	Free living	IDA
MIGS-14	Pathogenicity	Unknown	NAS
	Biosafety level	2	NAS
	Isolation	Soil of Ezzemoul Sabkha Lake	IDA
MIGS-4	Geographic location	Algeria	IDA
MIGS-5	Sample collection time	July 2012	IDA
MIGS-4.1	Latitude	35.856570	IDA
MIGS-4.1	Longitude	6.504890	IDA
MIGS-4.3	Depth	Unknown	NAS
MIGS-4.4	Altitude	800 m	IDA

^a^Evidence codes - IDA: Inferred from Direct Assay, TAS: Traceable Author Statement (i.e., a direct report exists in the literature), NAS: Non traceable Author Statement (i.e., not directly observed for the living, isolated sample, but based on a generally accepted property for the species, or anecdotal evidence). These evidence codes are from the Gene Ontology project [34]. If the evidence is IDA, then the property was directly observed for a live isolate by one of the authors or an expert mentioned in the acknowledgements.

**Figure 2:**
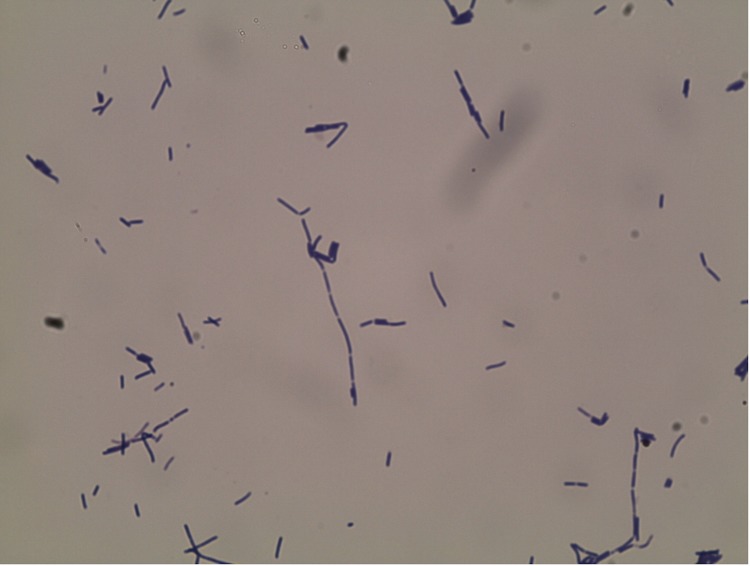
Gram stain of *Paucisalibacillus algeriensis* strain EB02^T^.

**Figure 3:**
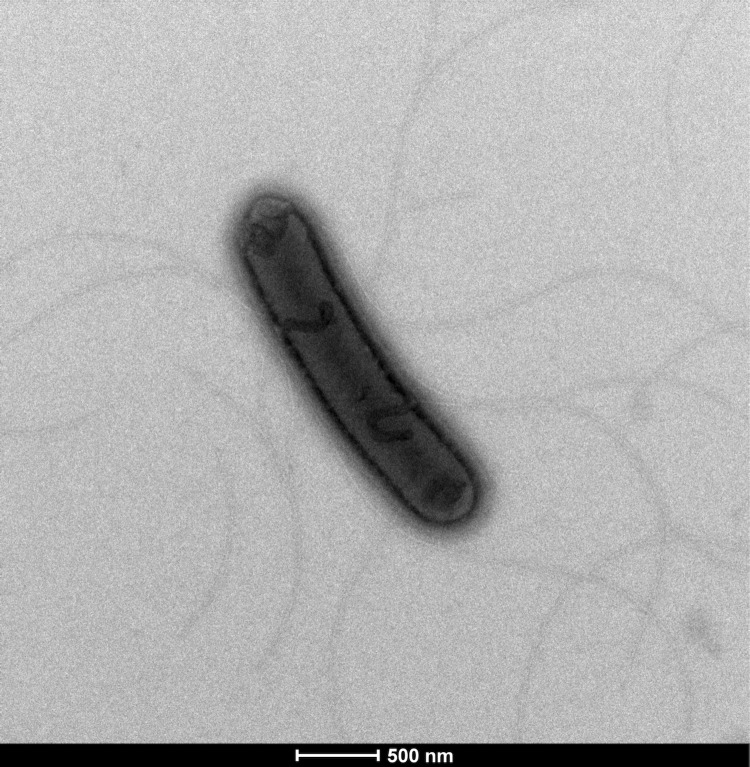
Transmission electron micrograph of *Paucisalibacillus algeriensis* strain EB02^T^ made using a Technai G^2^ Cryo (FEI) at an operating voltage of 200 kV. The scale bar represents 500 nm**.**

Six growth temperatures (25, 30, 37, 45, 50 and 55°C), ten pHs (5, 6, 6.5, 7, 7.5, 8, 8.5, 9, 10, 11) and nine NaCl concentrations (0, 2.5, 5, 7.5, 10, 15, 20, 25, 30%) were tested. Growth occurred between 25°C and 50°C, however the optimal growth was observed between 30°C and 37°C, the strain was able to grow at between 0% and 5% NaCl concentration and at pHs in the range of 6.5-9 (optimum at pH 7). After 24 h of aerobic incubation under optimal growth conditions on sheep blood agar (BioMerieux), strain EB02^T^ formed light beige, circular, slightly elevated colonies from 1mm to 2 mm in diameter. Growth of the strain was tested in anaerobic and microaerophilic atmospheres using GasPak EZ Anaerobe Pouch (Becton, Dickinson and Company) and CampyGen Compact (Oxoid) systems, respectively, and in an aerobic atmosphere, with or without 5% CO2. Growth was achieved under aerobic (with and without CO2) and microaerophilic conditions but no growth was observed under anaerobic conditions. Gram staining showed Gram positive rods (Figure 2). Cells grown on agar sporulate. A motility test was positive. The presence of peritrichous flagella and the size of cells were determined by negative staining transmission electron microscopy on a Technai G^2^ Cryo (FEI) at an operating voltage of 200 kV, the rods have a length ranging from 2.1 μm to 3.2 μm (mean 2.6 μm) and a diameter ranging from 0.4 μm to μm 0.6 (mean 0.5 μm) (Figure 3). Strain EB02^T^ exhibited catalase activity but oxidase activity was negative. Using the commercially available API ZYM system (BioMerieux), positive reactions were observed for alkaline phosphatase, esterase (C4), trypsin, α-glucosidase, and a weak positive reaction was observed for esterase lipase (C8); the other tests were negative. Using the API 50CH system (BioMerieux) according to the manufacturer’s instructions, a weak positive reaction was observed for D-glucose, D-fructose, N-acetylglucosamine, D-saccharose, amygdalin, esculin and salicin. The remaining tests were negative. Indole production, β-galactosidase, urease, and hydrolysis of gelatin and starch were negative, but nitrate reduction reaction was positive. *Paucisalibacillus algeriensis* was resistant to nalidixic acid, but susceptible to amoxicillin, nitrofurantoin, erythromycin, doxycycline, rifampicin, vancomycin, gentamicin, imipenem, trimethoprim-sulfamethoxazole, ciprofloxacin, ceftriaxone and amoxicillin-clavulanic acid.

When compared to other *Paucisalibacillus**,* *Ornithinibacillus**,* *Oceanobacillus* and *Virgibacillus*species [1,37-43], *Paucisalibacillus algeriensis* sp. nov. strain EB02^T^ exhibited the phenotypic differences detailed in (Table 2).

**Table 2:** Differential phenotypic characteristics between *Paucisalibacillus algeriensis* sp. nov. strain EB02^T^ and phylogenetically close members of family *Bacillacea*^†^.

**Characteristic**	1	2	3	4	5	6	7	8	9
Cell-diameter(µm)	0.4-0.6	0.5	0.4	0.4	0.3-0.5	0.5-0.6	0.5-0.6	0.6-0.8	0.6-0.8
Oxygen requirement	Strictly aerobic	Strictly aerobic	Strictly aerobic	Strictly aerobic	Strictly aerobic	Strictly aerobic	aerobic	Strictly aerobic	facultative anaerobic
Gram strain	+	+	+	+	+	+	+	+	V
NaCl range (%,w/v)	0-5	0-8	0.5-12	0-10	0-5	0.5-12.5	0-10	0-21	0-23
Motility	+	+	+	+	+	+	+	+	+
Endospore formation	+	+	+	+	+	+	+	+	+
**Production of**									
Alkaline phosphatase	+	na	na	na	+	na	na	na	+
Acid phosphatase	-	na	na	na	-	na	na	na	-
Catalase	+	+	+	+	+	+	+	+	+
Oxidase	-	-	+	+	+	-	+	v	+
Nitrate reductase	+	-	-	-	na	-	-	-	+
Urease	-	-	-	-	na	-	-	-	-
α-galactosidase	-	na	na	na	na	na	na	na	-
β-galactosidase	-	na	-	-	na	-	-	na	V
β-glucuronidase	-	na	+	+	na	na	na	-	-
N-acetyl-β-glucosaminidase	-	na	na	na	na	na	na	na	V
Indole	-	na	-	-	na	-	-	-	-
Esterase	+	na	-	-	+	na	na	+	+
Esterase lipase	w	na	w	w	+	na	na	w	+
Naphthyl-AS-BI-Phosphohydrolase	-	na	na	na	+	na	na	na	+
Leucine arylamidase	-	na	w	+	+	na	na	w	-
Cystine arylamidase	-	na	na	na	na	na	na	na	-
Valine arylamidase	-	na	na	na	na	na	na	na	-
**Utilization of**									
D-mannose	-	+	-	-	-	-	-	+	+
Amygdalin	w	+	-	-	+	na	na	-	na
L-Arabinose	-	-	-	-	-	-	+	-	-
Cellobiose	-	-	-	-	+	-	na	-	-
Lactose	-	+	-	-	na	+	na	-	+
D-xylose	-	+	-	-	+	na	+	-	-
D-Glucose	w	+	+	+	+	+	+	+	+
Mannitol	-	+	-	-	+	-	W	-	V(+)
Arabinose	-	-	-	-	-	-	na	-	-
L-Xylose	-	+	-	-	na	na	na	na	na
Glycerol	-	-	+	+	+	+	+	na	na
D-Galactose	-	+	-	-	na	-	na	-	+
**Hydrolysis of**									
Starch	-	+	-	-	+	+	na	-	-
Gelatin	-	+	+	+	-	-	-	+	+
**Habitat**	hyersaline soil	potting soil	sediment	pasteurized milk	dead ark clam	hypersaline water	wastewater	deep sea sediment	marine solar saltern

+: positive result, -: negative result, var: variable, w: weak positive result, na: data not available.

^†^Strains: 1, *Paucisalibacillus algeriensis* sp. nov. strain EB02^T^*;* 2, *Paucisalibacillus globulus*strain B22^T^; 3, *Ornithinibacillus californiensis*strain MB-9^T^; 4, *Ornithinibacillus bavariensis*strain WSBC 24001^T^; 5, *Ornithinibacillus scapharcae*strain TW25^T^; 6, *Ornithinibacillus halophilus*strain G8B^T^; 7, *Oceanobacillus caeni*strain S-11^T^*;* 8, *Oceanobacillus iheyensis*strain HTE831^T^ ; 9, *Virgibacillus halodenitrificans*strain SF 121^T^ .

**Figure 4:**
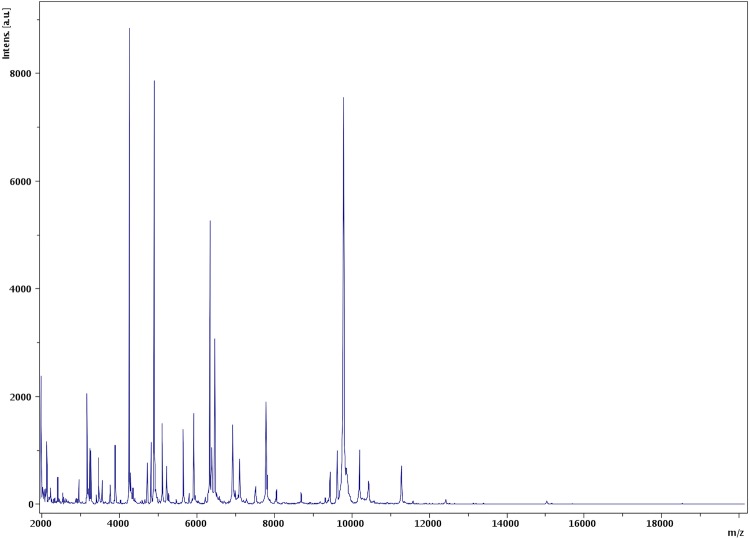
Reference mass spectrum from *Paucisalibacillus algeriensis* strain EB02^T^. Spectra from 12 individual colonies were compared and a reference spectrum was generated.

**Figure 5:**
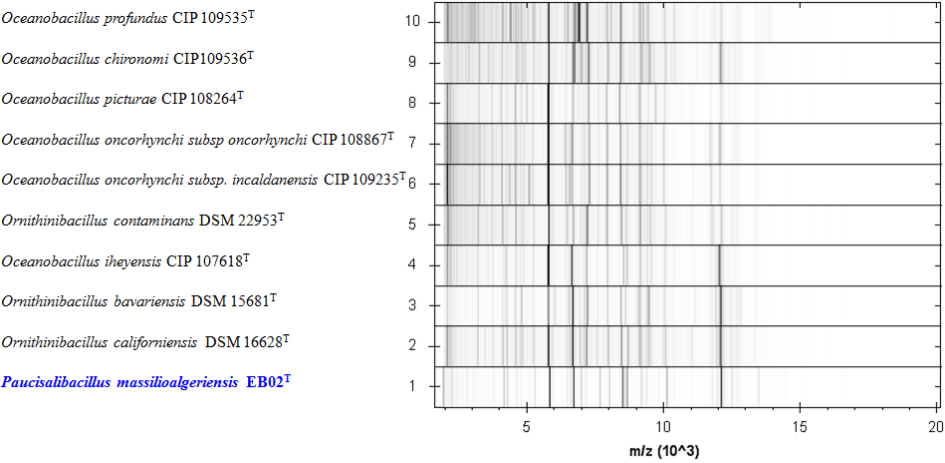
Gel view comparing *Paucisalibacillus algeriensis* EB02^T^ spectra with other members of *Ornithinibacillus** and* *Oceanobacillus*genera. The Gel View displays the raw spectra of all loaded spectrum files arranged as a pseudo-electrophoretic gel. The x-axis records the m/z value. The left y-axis displays the running spectrum number originating from subsequent spectra loading. The peak intensity is expressed by a grey scale scheme; more intense peaks are shown as darker shades of grey.

Matrix-assisted laser-desorption/ionization time-of-flight (MALDI-TOF) MS protein analysis was performed as previously described [12,44,45]. Briefly, strain EB02^T^ was cultivated on 5% sheep blood-enriched Columbia agar (BioMerieux) and incubated for 24 h at 30°C. Isolated bacterial colonies were picked, and then deposited as a thin film in 12 replicates on a MALDI-TOF steel target plate (Bruker Daltonics, Bremen, Germany). The plates were allowed to dry at room temperature. Each deposit was overlaid with 1.5 µl of matrix solution containing α-cyano-hydroxycinnamic acid (Sigma, Saint-Quentin Fallavier, France) saturated with 50% acetonitrile, 2.5% trifluoroacetic acid and high-performance liquid chromatography (HPLC)-grade water, and allowed to co-crystallize with the sample. Measurements were conducted using the Microflex LT spectrometer (Bruker Daltonics). Spectra were recorded in the linear positive ion mode over a mass range of 2 to 20 kDa. The acceleration voltage was 20 kV. Spectra were collected as a sum of 240 shots across a spot. The 12 EB02^T^ spectra were imported into the MALDI BioTyper software (version 3.0, Bruker) and analyzed by standard pattern matching (with default parameter settings) against 6,335 bacterial spectra supplemented by the spectra from *Ornithinibacillus californiensis* DSM 16628^T^, *Ornithinibacillus bavariensis* DSM 15681^T^, *Oceanobacillus iheyensis* CIP 107618^T^ which were the most closely related species on the basis of their 16S rRNA gene sequences, and *Ornithinibacillus contaminans* DSM 22953^T^, *Oceanobacillus oncorhynchi subsp incaldanensis* CIP 109235^T^, *Oceanobacillus picturae* CIP 108264^T^, *Oceanobacillus profundus* CIP 109535^T^, *Oceanobacillus oncorhynchi subsp. oncorhynchi* CIP 108867^T^, *Oceanobacillus chironomi* CIP 109536^T^, used as reference data in the BioTyper database. A score enabled the identification, or not, from the tested species: a score > 2 with a validated species enabled the identification at the species level, a score > 1.7 but < 2 enabled the identification at the genus level; and a score < 1.7 did not enable any identification. For strain EB02^T^, the scores obtained ranged from 1.0 to 1.4 thus suggesting that our isolate was a new species. We added the spectrum from strain EB02^T^ (Figure 4) to our database. Spectrum differences with those of *Ornithinibacillus* and *Oceanobacillus*related species are shown in (Figure 5).

## Genome sequencing information

### Genome project history

The organism was selected for sequencing on the basis of its phylogenetic position and 16S rDNA sequence similarity to other members of the genus *Paucisalibacillus*, and is part of a study of the microbial diversity of the hypersaline lakes in northeastern Algeria. It was the 2^nd^ genome of a *Paucisalibacillus*species and the first genome of *Paucisalibacillus algeriensis* sp. nov. The EMBL accession number is CBYO000000000 and consists of 23 contigs. Table 3 shows the project information and its association with MIGS version 2.0 compliance [46].

**Table 3:** Project information

**MIGS ID**	**Property**	**Term**
MIGS-31	Finishing quality	High-quality draft
MIGS-28	Libraries used	Nextera XT library
MIGS-29	Sequencing platform	Miseq-Illumina
MIGS-31.2	Sequencing coverage	80.57×
MIGS-30	Assemblers	SPAdes Genome assembler
MIGS-32	Gene calling method	Prodigal
	EMBL Date of Release	February 12, 2014
	EMBL ID	CBYO000000000
MIGS-13	Project relevance	Study of the microbial diversity of the hypersaline lakes in northeastern Algeria

### Growth conditions and DNA isolation

*Paucisalibacillus algeriensis* sp. nov strain EB02^T^, was grown aerobically on 5% sheep blood enriched Columbia agar at 30°C. Three petri dishes were spread and resuspended in a 2 ml sterile Eppendorf tube containing 1ml of TE buffer with acid-washed glass beads (diameter ≤106 µm, Sigma, Saint-Quentin Fallavier, France). Three cycles of shaking were performed using a FastPrep BIO 101 apparatus (Qbiogene, Strasbourg, France) for 15 sec at level 6.5 (full speed). Then, the supernatant was placed in a new tube along with one hundred μl of 10% SDS and 50 µl of Proteinase K (Qiagen GmbH, Hilden, Germany) and incubated over night at 56°C. The digested mixture was used to perform DNA extraction using the classical phenol-chloroform method. The quality of the DNA was checked on an agarose gel (0.8%) stained with SYBR safe.

### Genome sequencing

Genomic DNA of *Paucisalibacillus algeriensis* sp. nov. strain EB02^T^ was sequenced on the MiSeq instrument (Illumina, Inc, San Diego CA 92121, USA) with paired end and barcode strategies in order to be mixed with 7 others genomic projects constructed according the Nextera XT library kit (Illumina).

The gDNA was quantified by a Qubit assay with the high sensitivity kit (Life technologies, Carlsbad, CA, USA) to 43.7 ng/µL and dilution was performed to require 1ng of each small genome as input. The “tagmentation” step fragmented and tagged the DNA to generate an optimum insert size of 1.6-kb, validated on a high sensitivity Caliper-Perkin Elmer labchip (Caliper Life Sciences, Inc, Massachusetts, USA). Then limited cycle PCR amplification completed the tag adapters and introduce dual-index barcodes. After purification on Ampure beads (Life technolgies, Carlsbad, CA, USA), the libraries were then normalized on specific beads according to the Nextera XT protocol (Illumina). Normalized libraries are pooled into a single library for sequencing on the MiSeq. The pooled single strand library was loaded onto the reagent cartridge and then onto the instrument along with the flow cell. Automated cluster generation and paired-end sequencing with dual index reads was performed in a single 39-hour run with a 2x250 bp read length. Within this pooled run, the index representation was determined to be 7.1%. Total information of 2.4 G bases was obtained from a 320 K/mm2 density with 94.9% (5,757,000) of the clusters passing quality control (QC) filters. From the genome sequencing process, the 1,375,572 produced Illumina reads for *Paucisalibacillus algeriensis* EB02^T^ were filtered according to the read qualities and sizes using the fastq-mcf program (Ea-utils: command-line tools for processing biological sequencing data) [47]. 1,296,442 filtered read sequences were kept for genome assembly. The SPAdes Genome assembler (http://bioinf.spbau.ru/spades) was used with different kmer values (from 67 to 99, interval 4) and the best assembly result, with kmer value (n=95) producing 23 contigs with sizes from 1214-bp to 717,245-bp and average size of 174,207-bp, was retained for genome annotation.

### Genome annotation

Open Reading Frames (ORFs) were predicted using Prodigal [48] with default parameters. The predicted bacterial protein sequences were searched against the Clusters of Orthologous Groups (COG) databases and the GenBank database [49] using BLASTP. Ribosomal RNAs were found by using RNAmmer 2.1 server [50,51] and BLASTn against the GenBank database, whereas the tRNAScan-SE tool [52] was used to find tRNA genes. Transmembrane helices and lipoprotein signal peptides were predicted using phobius web server [53]. ORFans were identified if their BLASTP *E*-value was lower than 1e-03 for alignment length greater than 80 amino acids, if alignment lengths were smaller than 80 amino acids, we used an *E*-value of 1e-05. Artemis [54] was used for data management and DNA Plotter [55] was used for visualization of genomic features. To estimate the mean level of nucleotide sequence similarity at the genome level between *Paucisalibacillus algeriensis* sp nov. strain EB02^T^ and *Paucisalibacillus globulus*, *Ornithinibacillus scapharcae*, *Oceanobacillus iheyensis* and *Virgibacillus halodenitrificans*, we used the Average Genomic Identity of Orthologous gene Sequences (AGIOS) home-made software. Briefly, this software uses the Proteinortho software [56] to detect orthologous proteins between genomes compared two by two, then retrieves the corresponding genes and determines the mean percentage of nucleotide sequence identity among orthologous ORFs using the Needleman-Wunsch global alignment algorithm.

## Genome properties

**Figure 6:**
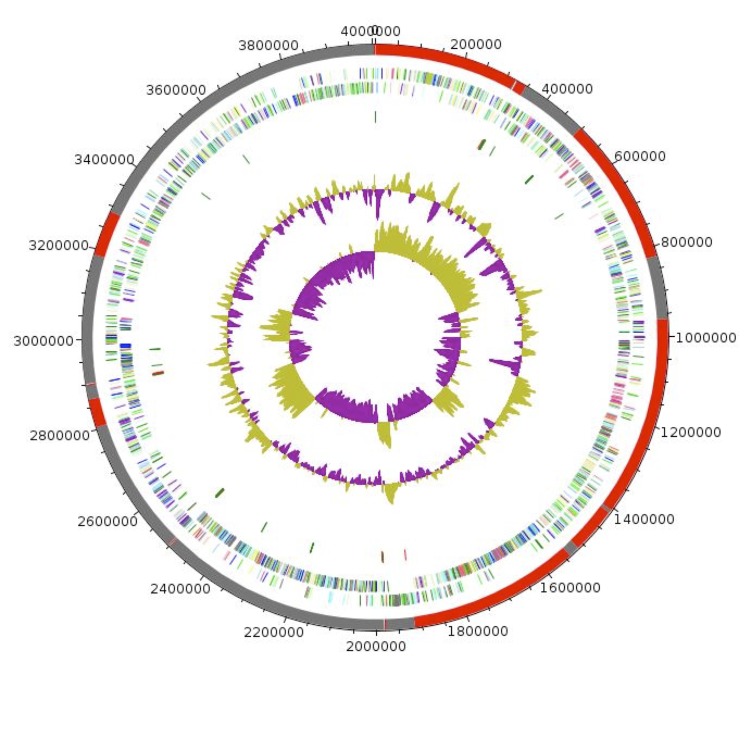
Graphical circular map of the chromosome. From outside to the center: Alternating red and grey showing the contigs, genes on the forward strand colored by COG categories (only genes assigned to COG), genes on the reverse strand colored by COG categories (only gene assigned to COG), RNA genes (tRNAs green, rRNAs red), GC content. The inner-most circle shows the GC skew, purple and olive indicating negative and positive values, respectively.

The genome is 4,006,766 bp long (1 chromosome but no plasmid) with 36% GC content (Figure 6 and Table 4). It is composed of 23 contigs. Of the 4,038 predicted genes, 3,956 were protein-coding genes, and 82 were RNAs (7 5S rRNA genes, 1 16S rRNA gene, 1 23S rRNA gene, and 73 tRNA genes). A total of 2,691 genes (68.02%) were assigned a putative function (by cogs or by NR blast), of which 179 were identified as ORFans (4.52%). The remaining genes were annotated as hypothetical proteins (821 genes, 20.75%). The distribution of genes into COGs functional categories is presented in Table 5. The properties and statistics of the genome are summarized in Tables 4 and 5.

**Table 4:** Nucleotide content and gene count levels of the genome

**Attribute**	Value	% of total^a^
Genome size (bp)	4,006,766	100
DNA coding region (bp)	3,426,186	85.51
DNA G+C content (bp)	1,442,326	36
Total genes	4,038	100
RNA genes	82	2.03
Protein-coding genes	3,956	97.96
Genes with function prediction	2,691	68.02
Genes assigned to COGs	2,551	64.48
Genes with peptide signals	450	11.37
Genes with transmembrane helices	1,067	26.97

^a^ The total is based on either the size of the genome in base pairs or the total number of protein coding genes in the annotated genome

**Table 5:** Number of genes associated with the 25 general COG functional categories

**Code**	**Value**	**%age**^a^	**Description**
J	173	4.37	Translation, ribosomal structure and biogenesis
A	0	0	RNA processing and modification
K	267	6.74	Transcription
L	138	3.48	Replication, recombination and repair
B	1	0.02	Chromatin structure and dynamics
D	32	0.80	Cell cycle control, mitosis and meiosis
Y	0	0	Nuclear structure
V	82	2.07	Defense mechanisms
T	130	3.28	Signal transduction mechanisms
M	143	3.61	Cell wall/membrane biogenesis
N	53	1.33	Cell motility
Z	0	0	Cytoskeleton
W	0	0	Extracellular structures
U	48	1.21	Intracellular trafficking and secretion
O	88	2.22	Posttranslational modification, protein turnover, chaperones
C	133	3.36	Energy production and conversion
G	184	4.65	Carbohydrate transport and metabolism
E	269	6.79	Amino acid transport and metabolism
F	80	2.02	Nucleotide transport and metabolism
H	76	1.92	Coenzyme transport and metabolism
I	100	2.52	Lipid transport and metabolism
P	176	4.44	Inorganic ion transport and metabolism
Q	83	2.09	Secondary metabolites biosynthesis, transport and catabolism
R	455	11.50	General function prediction only
S	265	6.69	Function unknown
-	1405	35.51	Not in COGs

^a^ The total is based on the total number of protein coding genes in the annotated genome

## Genome comparison with other genomes of *Paucisalibacillus**,* *Ornithinibacillus**,* *Oceanobacillus** and* *Virgibacillus*species

We compared the genome of *Paucisalibacillus algeriensis* strain EB02^T^ with those of *Paucisalibacillus globulus*strain B22^T^, *Ornithinibacillus scapharcae*strain TW25^T^, *Oceanobacillus iheyensis*strain HTE831^T^ and *Virgibacillus halodenitrificans*strain 1806^T^ (Table 6). The draft genome of *Paucisalibacillus algeriensis* (4.006Mb) is smaller in size than that of *Paucisalibacillus globulus*strain B22^T^ (4.24 Mb) but larger than those of *Oceanobacillus iheyensis*strain HTE831^T^, *Ornithinibacillus scapharcae*strain TW25^T^ and *Virgibacillus halodenitrificans*strain 1806^T^ (3.63, 3.84 and 3.92 Mb, respectively). *Paucisalibacillus algeriensis* has a higher G+C content (36%) than those of *Oceanobacillus iheyensis*strain HTE831^T^ and *Paucisalibacillus globulus*strain B22^T^ (35.7% and 35.8%, respectively) but lower than those of *Ornithinibacillus scapharcae*strain TW25^T^ and *Virgibacillus halodenitrificans*strain 1806^T^ (36.7% and 37.4%, respectively). *Paucisalibacillus algeriensis* has more predicted protein coding genes (3,956) than *Oceanobacillus iheyensis*strain HTE831^T^ and *Virgibacillus halodenitrificans*strain 1806^T^ (3,500 and 3,886, respectively) but fewer genes than *Ornithinibacillus scapharcae*strain TW25^T^ and *Paucisalibacillus globulus*strain B22^T^ (3,986 and 4,191, respectively). In addition, *Paucisalibacillus algeriensis* shared 2,591, 2,502, 2,002, and 2,083 orthologous genes with *Paucisalibacillus globulus*, *Ornithinibacillus scapharcae*, *Oceanobacillus iheyensis* and *Virgibacillus halodenitrificans*respectively.

**Table 6:** Genomic comparison of *Paucisalibacillus algeriensis* sp. nov. strain EB02^T^ with four other related species^†^.

**Species**	**Strain**	**Genome accession number**	**Genome size (Mb)**	**G+C content (%)**
*Paucisalibacillus algeriensis*	EB02^T^	CBYO000000000	4.01	36
*Paucisalibacillus globulus*	DSM 18846^T^	AXVK00000000	4.24	35.8
*Ornithinibacillus scapharcae*	TW25^T^	AEWH00000000	3.84	36.7
*Oceanobacillus iheyensis*	HTE831^T^	NC_004193	3.63	35.7
*Virgibacillus halodenitrificans*	1806^T^	ALEF00000000	3.92	37.4

**^†S^**pecies and strain names, genome accession numbers, sizes and G+C contents

The average nucleotide sequence identity of orthologous genes ranges from 69.15 to 83.06% among all of the genomes which were used for the comparison, and from 69.49 to 83.06% between *Paucisalibacillus algeriensis* and the other genomes (Table 6,. Table 7.), presenting a high sequence identity of orthologous genes with *Paucisalibacillus*genus (83.06%), thus confirming its new species status in the *Paucisalibacillus*genus.

**Table 7:** Genomic comparison of *Paucisalibacillus algeriensis* sp. nov. strain EB02^T^ with four other related species†.

Species	***P. ma***	*P. gl*	*Or. sc*	*O. ih*	*V. ha*
***Paucisalibacillus algeriensis***	**3,956**	2,591	2,502	2,002	2,083
*Paucisalibacillus globulus*	83.06	**4,191**	2,489	1,990	2,053
*Ornithinibacillus scapharcae*	75.99	75.96	**3,986**	1,928	1,997
*Oceanobacillus iheyensis*	69.49	69.51	69.15	**3,500**	1,875
*Virgibacillus halodenitrificans*	70.18	70.19	69.70	69.90	**3,886**

**†**Numbers of orthologous protein shared between genomes (above diagonal), average percentage similarity of nucleotides corresponding to orthologous proteins shared between genomes (below diagonal). Bold numbers indicate numbers of proteins per genome.

*Paucisalibacillus algeriensis* (*P. ma*), *Paucisalibacillus globulus* (*P. gl*), *Ornithinibacillus**scarpharcae* (*Or. sc*), *Oceanobacillus iheyensis* (*O. ih*), *Virgibacillus halodenitrificans* (*V. ha*)

## Conclusion

On the basis of phenotypic (Table 2), phylogenetic and genomic analyses (taxonogenomics) (Table 6, Table 7.), we formally propose the creation of *Paucisalibacillus algeriensis* sp. nov. that contains the strain EB02^T^. This strain has been found in a hypersaline lacustrine soil sample collected from Algeria.

### Description of *Paucisalibacillus algeriensis* sp. nov EB02^T^

*Paucisalibacillus algeriensis* (al.ge.ri.en’sis. N.L. masc.adj. *algeriensis*, of Algeria, where strain EB02^T^ was isolated). Strain EB02^T^ is a strictly aerobic Gram-positive rod, endospore-forming, motile by means of peritrichous flagella. Growth is achieved aerobically between 25°C and 50°C, but optimal growth was observed between 30°C-37°C. The strain was able to grow between 0% and 5% NaCl concentration and at pHs in the range of 6.5-9(optimum at pH 7). Growth is also observed under a microaerophilic atmosphere, however, no growth was observed under anaerobic conditions. After 24h growth on 5% sheep blood-enriched Columbia agar (BioMerieux) at 30°C, bacterial colonies were light beige, circular, slightly elevated and from 1 mm to 2 mm in diameter. Cells have a length ranging from 2.1 μm to 3.2 μm (mean 2.6 μm) and a diameter ranging from 0.4 μm to μm 0.6 (mean 0.5 μm).

Catalase activity was positive but oxidase activity was negative. Using the commercially available API ZYM system (BioMerieux), positive reactions were observed for alkaline phosphatase, esterase (C4), trypsin, α-glucosidase, and weak positive reaction was observed for esterase lipase (C8). The other tests were negative. Using the API 50CH system (BioMerieux) according to the manufacturer’s instructions, a weak positive reaction was observed for, D-glucose, D-fructose, N-acetylglucosamine, D-saccharose, amygdalin, esculin and salicin. The remaining tests were negative. Indole production, β-galactosidase, urease, hydrolysis of gelatin and starch were negative, but the nitrate reduction reaction was positive. *Paucisalibacillus algeriensis* was resistant to nalidixic acid, but susceptible to amoxicillin, nitrofurantoin, erythromycin, doxycycline, rifampicin, vancomycin, gentamicin, imipenem, trimethoprim-sulfamethoxazole, ciprofloxacin, ceftriaxone and amoxicillin-clavulanic acid. The G+C content of the genome is 36%.

The 16S rRNA and genome sequences are deposited in GenBank under accession number HG315680 and EMBL database under accession number CBYO000000000, respectively. The type strain EB02^T^ (= CSUR P858 = DSM 27335) was isolated from a soil sample from the margin of the hypersaline lake Ezzemoul Sabkha in the Oum-El-Bouaghi region of northeastern Algeria.
